# The Association of Kinesiophobia and Pain Catastrophizing with Pain-Related Disability and Pain Intensity in Obesity and Chronic Lower-Back Pain

**DOI:** 10.3390/brainsci11010011

**Published:** 2020-12-24

**Authors:** Giorgia Varallo, Emanuele Maria Giusti, Federica Scarpina, Roberto Cattivelli, Paolo Capodaglio, Gianluca Castelnuovo

**Affiliations:** 1 Department of Psychology, Catholic University of Milan, 20123 Milan, Italy; EmanueleMaria.Giusti@unicatt.it (E.M.G.); r.cattivelli@auxologico.it (R.C.); gianluca.castelnuovo@unicatt.it (G.C.); 2 Istituto Auxologico Italiano, IRCCS, Laboratorio di Psicologia, Ospedale S. Giuseppe, 28824 Piancavallo (Verbania), Italy; 3 “Rita Levi Montalcini” Department of Neurosciences, University of Turin, 10124 Turin, Italy; f.scarpina@auxologico.it; 4 Istituto Auxologico Italiano, IRCCS, U.O. di Neurologia e Neuroriabilitazione, Ospedale S. Giuseppe, 28824 Piancavallo (Verbania), Italy; 5 Istituto Auxologico Italiano, U.O. di U.O. Riabilitazione Osteoarticolare, Ospedale S. Giuseppe, 28824 Piancavallo (Verbania), Italy; p.capodaglio@auxologico.it; 6 Department of Surgical Sciences, Physical and Rehabilitation Medicine, University of Turin, 10121 Turin, Italy

**Keywords:** obesity, chronic lower-back pain, disability, pain perception, kinesiophobia, pain catastrophizing

## Abstract

Individuals affected by chronic lower-back pain and obesity have an increased risk of long-lasting disability. In this study, we aimed to explore the contribution of kinesiophobia and pain catastrophizing in explaining pain intensity and pain-related disability in chronic lower-back pain associated to obesity. A cross-sectional study on 106 participants with obesity and chronic lower-back pain was performed. We assessed pain intensity, pain disability, pain catastrophizing, and kinesiophobia levels through self-reporting questionnaire. Hierarchical regressions were performed to assess the role of pain catastrophizing and kinesiophobia on pain intensity and pain disability. According to the results, kinesiophobia, but not pain catastrophing, significantly explained both pain intensity and pain-related disability. Kinesiophobia might play a significant role in enhancing pain-related disability and the pain intensity in individuals with chronic lower-back pain and obesity. We encourage future studies in which beliefs and cognition towards pain might be a therapeutic target in interdisciplinary pain management interventions.

## 1. Introduction

Obesity is a public health issue of increasing importance [1] with substantial personal, community, and financial burdens globally [1]. It is strongly related to lower levels of physical and emotional well-being [2], and higher levels of mobility disability [3], especially when associated with other clinical conditions, including pain-related conditions [4]. Specifically, obesity is a risk factor for chronic lower-back pain (CLBP) [5], a clinical condition characterized by persistent pain (more than 3 months), in absence of a recognizable mechanical origin [6]. Obesity and CLBP share a high comorbid burden [5,7]. Affected individuals face profound functional and physical limitations [8]. Moreover, they generally report a high degree of pain-related disability in daily life [9]. The relationship between obesity and pain is complex and multifactorial [10]; evidence indicates that it might involve a combination of systemic inflammation, musculoskeletal overload, and autonomic dysregulation [10,11,12]. Also, psychological factors have to be taken into account as contributing to the issue [13,14].

There is an ongoing debate about which individual components might affect pain intensity and the perceived level of disability. Pain perception seems to be influenced by several factors, including emotions and cognition towards pain symptoms [15]. Within this debate, growing relevance was assumed by the fear-avoidance model [16,17], according to those individuals who experience acute pain may become trapped in a vicious circle of chronic disability and suffering, because of individual cognitive and affective responses towards pain [18,19,20]. Also, maladaptive behavioral coping strategies (i.e., avoidance behaviors) seem to be related to cognitive and affective responses towards pain [21], such as catastrophizing and kinesiophobia. Pain catastrophizing is defined as a set of exaggerated and negative cognitive-emotional responses to actual or anticipated painful sensations [21], leading individuals to magnify or exaggerate the threat of pain stimuli and to feel helpless because of it [22]. Kinesiophobia is an excessive, irrational, and debilitating fear towards physical movement and activity resulting from a feeling of vulnerability due to a painful injury or fear of reinjury [23,24]. Even though these behaviors may be adaptive in the case of acute pain, nevertheless they seem to be dysfunctional when the painful condition becomes chronic. Indeed, they might contribute to perpetuating physical activity aversion, thereby worsening mobility, pain severity, aggravating disability, and lowering the pain threshold [10,13,25]. Vincent and colleagues [26] suggested that, in the case of CLBP, those individuals affected by obesity reported higher levels of kinesiophobia in comparison with individuals with a healthy weight. Moreover, higher levels of kinesiophobia predicted higher levels of perceived disability [26]. Similarly, high levels of pain catastrophizing were reported to be associated with higher levels of pain related disability and pain intensity [27]. Despite these preliminary results, research about the role of kinesiophobia and pain catastrophizing in relation to pain intensity and pain-related disability in individuals with CLBP and obesity is still in its infancy. 

To the aim to clarify this issue, in the present cross-sectional study, we aimed to assess the role of kinesiophobia and pain catastrophizing as predictive factors for pain-related disability and pain intensity in a sample of individuals affected by both obesity and CLBP.

## 2. Materials and Methods

A cross-sectional study was performed. One hundred and six individuals were consecutively recruited, from 1 September 2018 to 31 July 2019, at the beginning of a month-long hospitalization for weight loss and physical therapy, at the Istituto Auxologico Italiano, U.O. di Riabilitazione Osteoarticolare, Ospedale S. Giuseppe, Piancavallo, Italia. Data were collected during the first week of diagnostic assessment, prior to the start of physical therapy and nutritional rehabilitation for weight loss. A priori sample size was estimated using G.Power (version 3.1.9.4) [28] setting a medium effect size (0.15), an alpha of 0.05 and a power of 0.80, resulting in 92 participants. Inclusion criteria were: age in years ≤70; obesity, according to a body mass index (i.e., BMI computed as the weight in kilograms divided by the square of height in meters, (kg/m^2^) ≥ 30) [29]; CLBP, defined as low back pain duration > 3 months [30].

Exclusion criteria were physical or mental inability to provide signed informed consent; acute LBP or LBP duration < 3 months; specific LBP conditions (diagnosis of fracture, neoplasia, bone metastasis, stenosis); neurogenic or radicular condition; neurological disease; diagnosis of another disease that may explain lumbar pain; post-operative pain.

### 2.1. Measures

The main outcomes were assessed as follows:

- Numerical Pain Rating Scale (NPRS) was used to assess the individual’s perception of pain intensity, through an 11-point scale (anchors of 0 = no pain, 10 = worst possible pain). The NPRS measure is an established, well-accepted outcome for chronic pain conditions [31]. This scale is a reliable and valid measurement instrument for assessing pain intensity [31], also in the case of chronic conditions [30].
- The Italian validation of the Roland–Morris Disability Questionnaire (RMDQ) [32], was used to assesses LBP-related physical disability. The RMDQ includes 24 dichotomous items covering daily tasks that participants have difficulty performing due to LBP. A total RMDQ score ranging from 0 to 24 can be computed. The RMDQ, in its Italian validation, showed levels of reliability and validity comparable to the original version [32]. Higher levels of pain-related disability are reflected by higher RMDQ scores.

We used the following questionnaire to measure the predictors:

- The Italian validation of Tampa Scale of Kinesiophobia (TSK) [33] was used to assess the level of kinesiophobia. The TSK includes 13 items on a four-point Likert scale ranging from “strongly disagree” to “strongly agree” [34]. The TSK has been validated for use in individuals affected by chronic LBP [16]. The Italian version of the TSK shows a good factorial structure and acceptable psychometric properties [33]. Total Score ranges from 17 to 68, with higher scores suggesting higher levels of kinesiophobia [33].
- The Italian validation of pain catastrophizing scale (PCS) [35] was used to assess the level of catastrophic thinking about pain. The PCS is composed of 13-items on a five-point Likert scale (from 0 = “not at all” to 4 = “all the time”), developed for both clinical and non-clinical populations. The Italian version shows good psychometric properties in agreement with the original version [35]. Total score ranges from 0 to 52; with higher scores suggesting higher levels of pain catastrophizing [35].

### 2.2. Statistical Analysis

Counts and percentages were used to describe categorical variables, whereas means and standard deviations were used to describe continuous variables. To evaluate the contribution of TSK score and PCS score to the variance of NPRS score and RMDQ score, two independent multiple hierarchical regression analyses were performed considering NPRS score (model 1) and RMDQ score (model 2) as dependent variables. In both models, confounding factors that likely affect main outcomes (pain intensity and pain-related disability), were included in the first block; PCS score and TSK score were included in the second block. Confounding factors in model 1 were: gender, age and BMI [36,37]. Confounding factors in model 2 were: gender, age, BMI [36,37] and NPRS scores [38]. ΔR^2^ was used to evaluate the additional amount of variance in the dependent variables that was accounted for by the variables included in the second block. The analyses were performed using Jamovi (version 1.2) [39]. *p*-Values of less than 0.05 were considered statistically significant.

## 3. Results

### 3.1. Participants Characteristics

The sample consisted of 68 women and 38 men. The description of demographical and clinical factors, as well as the questionnaires scores, are reported in Table 1.

### 3.2. Predictors of Pain Intensity

The full model of gender, age, BMI, PCS score, and TSK score as predictors and NPRS scores as the dependent variable was statistically significant, R^2^ = 0.198, F (5,100) =4.94, *p* < 0.001. The inclusion of the PCS score and the TSK score explained about 18% additional variance (ΔR^2^ = 0.177), compared to the first block including only the confounding factors. Only the TSK score significantly predicted pain intensity (Table 2).

### 3.3. Predictors of Pain-Related Disability

The full model of gender, age, BMI, NPRS score, PCS score, and TSK score as predictors and RMDQ score as the dependent variable was statistically significant, R^2^ = 0.339, F (6, 99) = 8.46, *p* < 0.001. The inclusion of the PCS score and the TSK score explained about 10% additional variance (ΔR^2^ = 0.102), compared to the first block including only the confounding factors. It was found that TSK score significantly predicted the RMDQ score (Table 3).

## 4. Discussion

The study aimed to verify the role of kinesiophobia and pain catastrophizing in explaining pain intensity and pain-related disability in a sample of individuals with obesity and CLBP. According to the results, we observed that kinesiophobia, but not pain catastrophizing, significantly explained both the subjective levels of pain intensity and pain-related disability.

Kinesiophobia is a key component of the fear-avoidance model of chronic pain [19], and its pivotal role, in explaining pain intensity and disability, was previously supported in individuals affected by obesity and CLBP [40]. Kinesiophobia results from the misinterpretation of painful bodily sensations as a sign of serious injury. Consequently, individuals might tend to avoid pain-related movements driven by the emotion of fear, increasing disuse, and disability [41].

We might assume that obesity plays a central role in explaining the important role of kinesiophobia in our sample. Because of the associated pathologies like respiratory difficulty, greater movement difficulties and discomforts [42], individuals affected by obesity may develop greater aversion and fear of movement. Supporting this explanation, it has been previously reported by Vincent et al. [26] that adults affected by severe obesity and CLBP reported higher levels of kinesiophobia compared to their normal-weight counterparts [26]. Also, the same authors examined the relationships between LBP, kinesiophobia and disability in overweight, older adults with LBP [40], observing that the TSK score are significant predictors of LBP severity and perceived disability [40]. Interestingly, our study and those of Vincent and colleagues shared similar results, even though different ages were considered: indeed, although Vincent and colleagues studied an elderly population, our participants showed a wider age range.

Conversely, we reported that pain catastrophizing was not significantly associated with pain intensity and pain-related disability; these results seemed in disagreement with previous evidence [20,38,43,44]; nevertheless, previous research did not focus specifically on the population of individuals with obesity. Differently, our sample is composed predominantly of individuals with moderate and severe obesity.

This study has several limitations. First, it is a cross-sectional study: although a significant association has been found between kinesiophobia and both pain severity and pain-related disability, it is not possible to establish a causal relationship. Longitudinal studies will be necessary to confirm the hypothesis that maladaptive cognitions influence the development and maintenance of pain and disability in individuals with CLBP and obesity. Secondly, the sample was composed exclusively of hospitalized individuals, which may induce a selection bias: thus, generalization to patients from different settings should be carefully done. Individuals with CLBP and obesity might have different coping mechanisms about pain in comparison with healthy weight individuals, that have not been considered in this study. For example, we have not included in the confounding factors the drug regimen of the subjects enrolled. It has been previously reported [26] that individuals with obesity reported a higher consumption of narcotic use to control pain symptoms compared to their normal-weight counterparts, suggesting that, in this specific population, drug use may be an avoidance coping strategy. Also, we did not assess the presence of emotional eating. Nevertheless, if in our sample emotional eating as a coping strategy of pain was predominant, this pattern would lead to positive energy balance and, ultimately, weight gain, obesity, and increased pain and disability, as suggested by a previous study [45].

On the other hand, this article has several strengths, which include the use of validated, reliable survey instruments, as well as an adequate sample size, and the fact that it presents data on a population on which there is still little research. Moreover, we assessed a clinical population (i.e., individuals with CBLP and obesity), about which very little evidence is reported in the literature.

Future research should investigate the role of kinesiophobia on pain intensity and pain-related disability in individuals with obesity, while also taking into account the role of physical exercise and physical therapy. Finally, given the aim of the study, in this work we did not take into account what was the primary medical condition (i.e., medical, psychological, socio-economical, and so on) which might have been contributed to obesity. In the future, it could be interesting to consider the determinants of obesity and their possible influence on kinesiophobia, pain intensity, and pain-related disability.

## 5. Conclusions

Psychological factors play an important role in pain management. Our study emphasizes the role of kinesiophobia in pain intensity and pain-related disability. This result could be a helpful tool to identify those individuals most at risk of developing a self-perception of negative functional ability. This might be crucial in designing rehabilitative programs to manage pain effectively. Moreover, kinesiophobia can be a useful therapeutic target to be included in interdisciplinary pain management interventions in individuals with obesity and chronic LBP.

## Figures and Tables

**Table 1:** Demographic and clinical characteristics of the sample (*n* = 106)

	*n* (%)	Mean ± sd
Gender Female Male	68 (64.2) 38 (35.8)	
Age		57.1±9.67
Body Mass Index (kg/m^2^)		39.8 ± 5.58
Numeric Pain Rating Scale (NPRS)		6.15 ± 2.45
Roland Morris Disability Questionnaire (RMDQ)		11.33 ± 6.74
Tampa Scale of Kinesiophobia (TSK)		29.9 ± 7.96
Pain Catastrophizing Scale (PCS)		23.5 ± 11.1

Note. Descriptive statistics are frequencies and percentages for categorical variables and means ± sd for continuous variables. NPRS range: 0–10, RMDQ range: 0–24, TSK range: 11–44 PCS range: 0-52. Abbreviations: sd = standard deviation.

**Table 2:** Multivariable linear regression model examining the independent effect of demographic features and psychological components of kinesiophobia (TSK score) and pain catastrophizing (PCS score) on pain intensity (NPRS score)

	B	95% CI	*p*-Value
Block 1: Confounding Factors			
Age	−0.009	−0.06–0.04	0.700
Gender	−0.524	−1.44–0.39	0.259
BMI	−0.038	−0.04–0.12	0.346
Block 2: Psychological variables			
Tampa Scale of Kinesiophobia (TSK)	0.126	0.07–0.18	<0.001 *
Pain Catastrophizing Scale (PCS)	0.010	−0.03–0.05	0.528

Note. Abbreviations: B = unstandardized beta; CI = confidence interval. TSK = Tampa Scale of Kinesiophobia; PCS = Pain Catastrophizing Scale; NPRS = Numeric Pain Rating Scale. * *p* < 0.05

**Table 3:** Multivariable linear regression model examining the independent effect of demographic features and psychological components of kinesiophobia (TSK score) and pain catastrophizing (PCS score) on pain-related disability (RMDQ score)

	B	95% CI	*p*-Value
Block 1: Confounding Factors			
Age	0.084	−0.03–0.20	0.162
Sex	−1.424	−3.74–0.89	0.226
BMI	−0.06	−0.26–0.14	0.555
Numeric Pain Rating Scale (NPRS)	0.741	0.24–1.23	0.004 *
Block 2: Psychological factors			
Tampa Scale of Kinesiophobia (TSK)	0.298	0.13–0.46	<0.001 *
Pain Catastrophizing Scale (PCS)	0.008	−0.09–0.11	0.874

Note. Abbreviations: B = unstandardized beta; CI = confidence interval. TSK = Tampa Scale of Kinesiophobia; PCS = Pain Catastrophizing Scale; RMDQ = Roland–Morris Disability Questionnaire. * *p* < 0.05

## Data Availability

The data presented in this study are available on request from the corresponding author. The data are not publicly available due to their containing information that could compromise the privacy of research participants.
